# Immunostimulatory nucleic acid nanoparticles establish antiviral state to inhibit viral infection and replication

**DOI:** 10.1128/jvi.01627-25

**Published:** 2026-02-03

**Authors:** Martin Panigaj, Cassandra Catacalos-Goad, Anh Ha, Valery Z. Grdzelishvili, Kirill A. Afonin

**Affiliations:** 1 Chemistry and Nanoscale Science Program, Department of Chemistry, University of North Carolina at Charlotte, Charlotte, North Carolina, USA; 2 Department of Biological Sciences, University of North Carolina at Charlotte, Charlotte, North Carolina, USA

**Keywords:** nucleic acid nanoparticles, innate immunity, interferon response, pattern recognition receptors, antiviral state, vesicular stomatitis virus, Sendai virus, respiratory syncytial virus

## Abstract

Establishing a rapid, broad-spectrum antiviral state in human cells offers a promising strategy to combat viral infections, especially when vaccines or pathogen-specific treatments are unavailable. Here, we evaluate immunostimulatory nucleic acid nanoparticles (iNANPs), identified as potent innate immune activators, for their ability to induce protective antiviral states. By mimicking pathogen-associated molecular patterns, iNANPs engage intracellular pattern recognition receptors to stimulate type I and III interferon responses. We tested iNANPs for antiviral efficacy against a replication-incompetent lentiviral vector pseudotyped with vesicular stomatitis virus (VSV) G protein, as well as replication-competent viruses, including VSV, Sendai virus (SeV), and respiratory syncytial virus (RSV). These viruses vary in their mechanisms of innate immune activation and evasion, providing a robust system to assess iNANP activity. Our results demonstrate that iNANPs dramatically restrict viral infection via induction of a robust IFN response, establishing an antiviral state that impairs replication of all tested viruses. This study highlights the potential of iNANPs as a broad-spectrum antiviral prophylactic platform.

Vaccines and antiviral medications are essential for controlling viral diseases; however, when such treatments are unavailable, innovative strategies are needed to induce an antiviral state in human tissues and prevent infections in high-risk settings or during seasonal outbreaks. Inducing such a state relies on activating innate immunity, the body’s first line of defense against infection. Innate immunity can be activated by immunostimulatory nucleic acid nanoparticles (iNANPs), which combine the precise molecular programmability of RNA and DNA with the broad functional versatility inherent to these essential biopolymers. The highly modular design and tunable physicochemical properties of nucleic acids enable the construction of iNANPs with defined architectures and compositions that can be tailored to engage specific innate immune sensors. This same modularity allows a seamless “plug-and-play” integration of diverse therapeutic nucleic acids, such as antisense oligonucleotides, siRNAs, aptamers, as well as other therapeutics and small molecules, directly into the iNANP structure (1–3). The embedded functionalities further expand the therapeutic potential of iNANPs beyond the innate immune stimulation. For instance, iNANPs assembled from six RNA or DNA strands can be easily decorated with six different functional moieties that can be simultaneously delivered to diseased cells (1, 4). By embedding six different siRNAs targeting various HIV-1 genes into hexagonal RNA ring NANPs, we previously achieved efficient suppression of viral production (1). A wide range of antiviral siRNAs and aptamers is now available, further broadening the possibilities for future designs (5, 6). Additionally, the choice of delivery carrier offers another modality that affects NANP behavior *in vivo*, affecting their stability, circulation lifespan, biodistribution, interactions with the immune system, cellular uptake, and intracellular localization (7–11).

The speed of virus replication is one of the critical factors determining disease outcome (12, 13). A rapid early innate immune response is essential to limiting viral load and preventing dissemination before adaptive immunity becomes fully activated. Pre-priming or boosting these innate pathways can provide broader and more immediate antiviral protection. The rapid activation of the innate immune system defines effective antiviral preparedness. This response begins with the triggering of innate immune sensors, followed by the expression of interferons and interferon-stimulated genes (ISGs), which suppress viral replication and establish an antiviral state in surrounding cells. The process is initiated by the detection of pathogen-associated mole cular patterns (PAMPs) by host pattern recognition receptors (PRRs), including Toll-like receptors (TLRs) and retinoic acid-inducible gene I (RIG-I)-like receptors. Activation of these pathways triggers the production of interferons (IFNs) and other cytokines, which inhibit viral entry, replication, and spread, while enhancing the adaptive immune system’s ability to clear infection (14).

Different PRRs recognize distinct molecular signatures: RIG-I senses short 5-tri- or diphosphorylated RNAs; MDA5 responds to long double-stranded (ds) RNA; TLR3 detects endosomal dsRNA; and TLR7/8 recognize single-stranded RNAs with defined sequence features. Targeting PRRs with specific agonists can enhance the cells’ antiviral state, offering a promising strategy for therapeutic intervention.

Nucleic acids can be rationally designed to stimulate the innate immune system by mimicking PAMPs recognized by specific PRRs (15). Furthermore, the programmability of nucleic acids enables their rational design and bottom-up self-assembly into multi-stranded architectures with predictable physicochemical and biological properties, including regulated immunorecognition. These immunostimulatory nucleic acid nanoparticles, or iNANPs, encode compositional parameters, such as shape, size, and chemical composition, that allow for precise interactions with selected PRRs to tailor elicitation of specific immune responses (2, 16, 17).

Our previous research extensively evaluated various iNANPs and identified three-dimensional RNA cubes assembled from six *in vitro*-transcribed short RNA strands as the most potent immunostimulatory nanostructures (16). We also discovered that the choice of delivery carrier constitutes an important variable that dictates iNANP recognition by PRRs and innate immune activation (8, 10).

To test if iNANPs can efficiently activate a rapid, broad-spectrum antiviral state in human cells, we examined the effects of RNA cubes on replication-incompetent second-generation lentiviral vector (LV) pseudotyped with vesicular stomatitis virus (VSV) G protein, as well as a diverse panel of replication-competent viruses, including VSV, Sendai virus (SeV), and respiratory syncytial virus (RSV). All tested viruses carry a GFP transgene to track viral infection.

Each virus was selected for its unique biological features and well-established utility in immunological research, particularly concerning type I IFN responses. Our LV is a retroviral vector system based on the human immunodeficiency virus developed to mediate stable *in vivo* gene transfer (18). VSV is a prototypic nonsegmented negative-sense RNA virus frequently used as a model for studying RIG-I-mediated innate immune signaling. VSV (a rhabdovirus) is a significant veterinary pathogen. Its rapid replication and strong induction of ISGs make it an excellent system for examining early antiviral responses (19, 20). Uniquely, VSV inhibits host gene expression through its matrix protein, which disrupts nuclear-cytoplasmic RNA transport, thereby dampening the immune responses (21–23).

RSV (a pneumovirus) is a clinically significant and highly prevalent human pathogen (24). RSV employs potent mechanisms to antagonize host IFN responses primarily through its nonstructural proteins NS1 and NS2, which degrade or inhibit key signaling molecules, such as STAT2 and RIG-I, thereby suppressing type I IFN production and downstream signaling (25).

Sendai virus (SeV), a parainfluenza virus in the paramyxovirus family that is not a human pathogen, is closely related to human parainfluenza viruses and widely used as a model for studying innate immune responses to respiratory viruses (26). It is known for its strong activation of RIG-I and MAVS-dependent signaling pathways, making it a powerful tool for probing the mechanisms of viral RNA recognition (27). SeV can antagonize innate immunity via its C proteins, which inhibit IFN signaling by targeting STAT1 and interfering with RIG-I activation (27).

By including LV, VSV, RSV, and SeV in the current experimental design, we ensured a broad evaluation of antiviral properties and host responses to tested iNANPs. Also, the inclusion of multiple viruses helps differentiate virus-specific effects from generalizable antiviral resistance phenotypes, providing a robust framework to assess the breadth and specificity of iNANP-induced antiviral states.

While induction of the cellular immune system with simple nucleic acid PRR agonists has proven to be an effective therapeutic strategy against viral infections and cancers (28, 29), our findings further demonstrate that iNANPs represent a programmable, nucleic acid-based immunotherapeutic platform for broad-spectrum antiviral prophylaxis and hold strong promise as next-generation adjuvants against infectious diseases.

## RESULTS

### Rapid innate immune activation by iNANPs depends on time of exposure

Rapid expression of type I and III IFNs in response to viral expression is a crucial step in cell defense for enacting an antiviral state and development of adaptive immunity (30). Induction of an antiviral state prior to infection, thus, has prophylactic potential when the causative agent of infection is unknown, but virus spread is highly probable in places with high human population density, during long travels, or traveling to exotic destinations.

**FIG 1:**
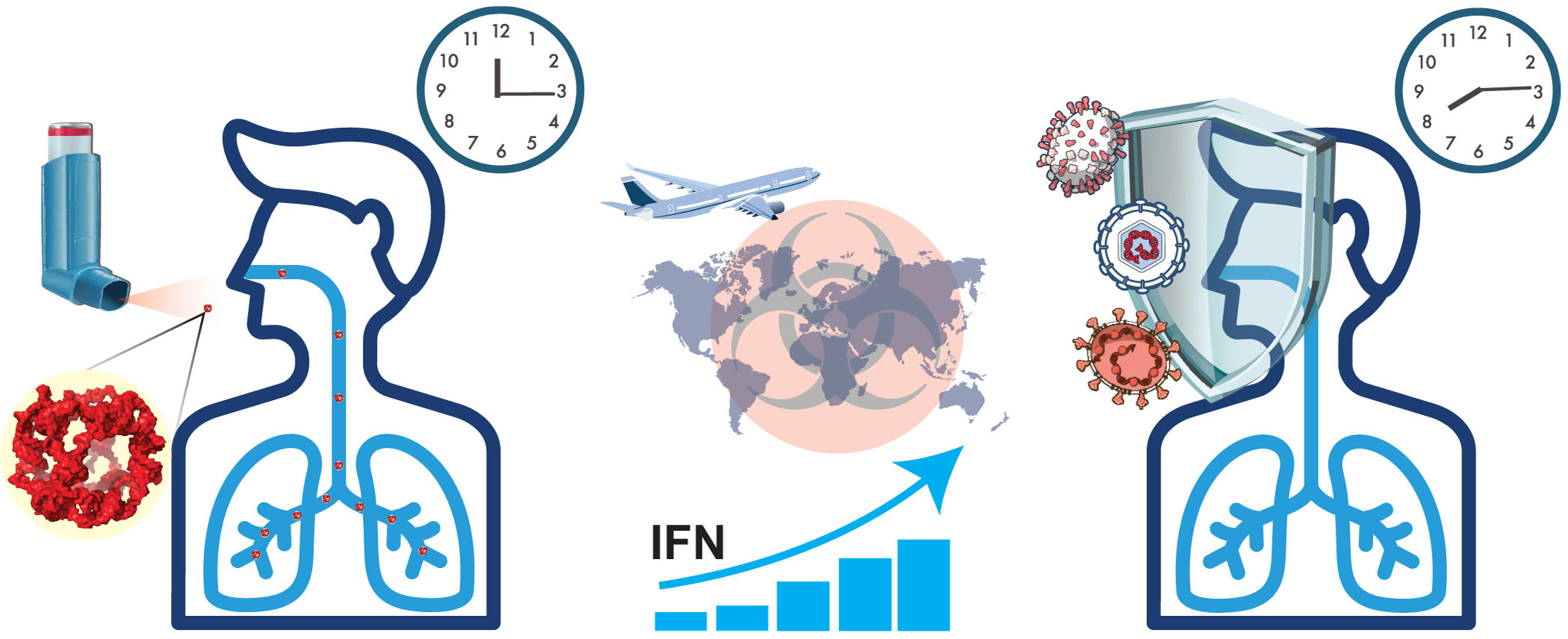
Proposed prophylactic application of iNANPs.

**FIG 2:**
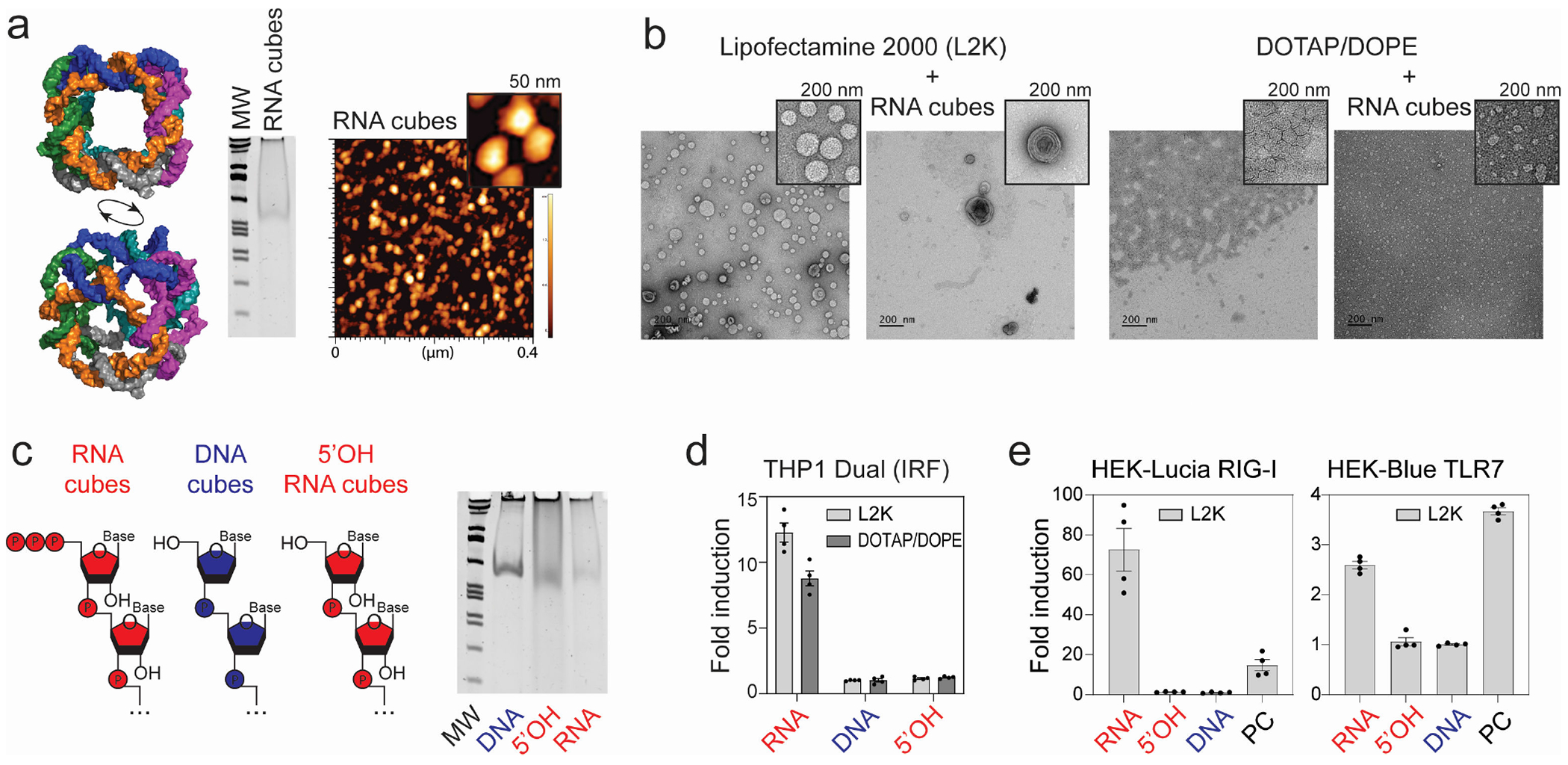
Confirmation of RNA cube assembly and comparison of their immunostimulatory properties to DNA and 5′OH RNA cubes in reporter cell lines. (a) 3D models of RNA cube iNANPs used in this study alongside representative native PAGE and AFM results confirming their assembly. (b) Representative TEM images of delivery carriers with and without iNANPs. (c) Comparison of three distinct NANPs in THP1 dual reporter cell lines via the IRF pathway (error bars represent mean ± SEM, *n* = 4). Nomenclature of iNANP variants: RNA—RNA cubes with six 5′-triphosphate groups; 5′OH—RNA cubes with six hydroxyl groups at the 5′ ends; and DNA—DNA cubes. (e) Confirmation of assembly of cube NANPs by native PAGE. (d) Comparison of RNA, 5′OH, and DNA cubes to induce expression of luciferase or SEAP through activation of RIG-I or TLR7 PRRs in reporter cell lines HEK-Lucia or HEK-Blue TLR7, respectively. *n* = 4, ±SEM.

In our previous studies, we showed that six-stranded RNA cubes induce IFNs and reduce intracellular bacterial burden of *S. aureus* in infected bone cells via stimulation of IFN-β expression (31). In this study, we examined whether RNA cubes with proper carrier formulation can serve as a novel prophylactic for respiratory virus infections (Fig. 1). The six individual RNA strands were transcribed *in vitro* and designed to self-assemble into RNA cubes, and successful formation was confirmed by native polyacrylamide gel electrophoresis (native-PAGE) and atomic force microscopy (AFM), as shown in Fig. 2a and c.

Since the choice of carrier can influence the immunorecognition of iNANPs, we complexed our RNA cube iNANPs with two alternative delivery reagents, Lipofectamine 2000 (L2K) and DOTAP/DOPE (Fig. 2b). While cationic-lipid transfection reagent L2K is considered a “gold standard” for experimental DNA and RNA delivery, DOTAP/DOPE is frequently adopted in clinical studies for mRNA vaccine delivery (32–35).

To assess the impact of iNANP composition on immunostimulatory potential, we used reporter cell lines to compare three cube variants: RNA cubes assembled from transcribed strands carrying 5′-triphosphates (“RNA”), synthetic RNA cubes lacking 5′-triphosphates (“5′OH”), and DNA cubes (“DNA”) assembled from six DNA strands of identical sequences.

As shown in Fig. 2d, RNA cubes activate the IRF pathway in THP-1 Dual cells, whereas 5′OH and DNA cubes failed to trigger significant activation. RIG-I was identified as the primary PRR recognizing RNA cubes, with strong luciferase induction observed in HEK-Lucia RIG-I reporter cells, while 5′OH and DNA cubes induced minimal activation (Fig. 2e). Interestingly, RNA cubes also activated the TLR7-dependent NF-κB pathway, whereas DNA and 5′OH cubes did not (Fig. 2e).

Since RNA cubes were the most potent activators of RIG-I and TLR7-dependent pathways, we selected A549 lung carcinoma epithelial cells as a model system to investigate the antiviral state induced by these iNANPs. A549 cells derived from human alveolar epithelial cells are widely used as an *in vitro* model for respiratory viral infections. They support the entry and replication of many clinically relevant viral pathogens and retain an intact innate immune system capable of producing cytokines in response to infection.

**FIG 3:**
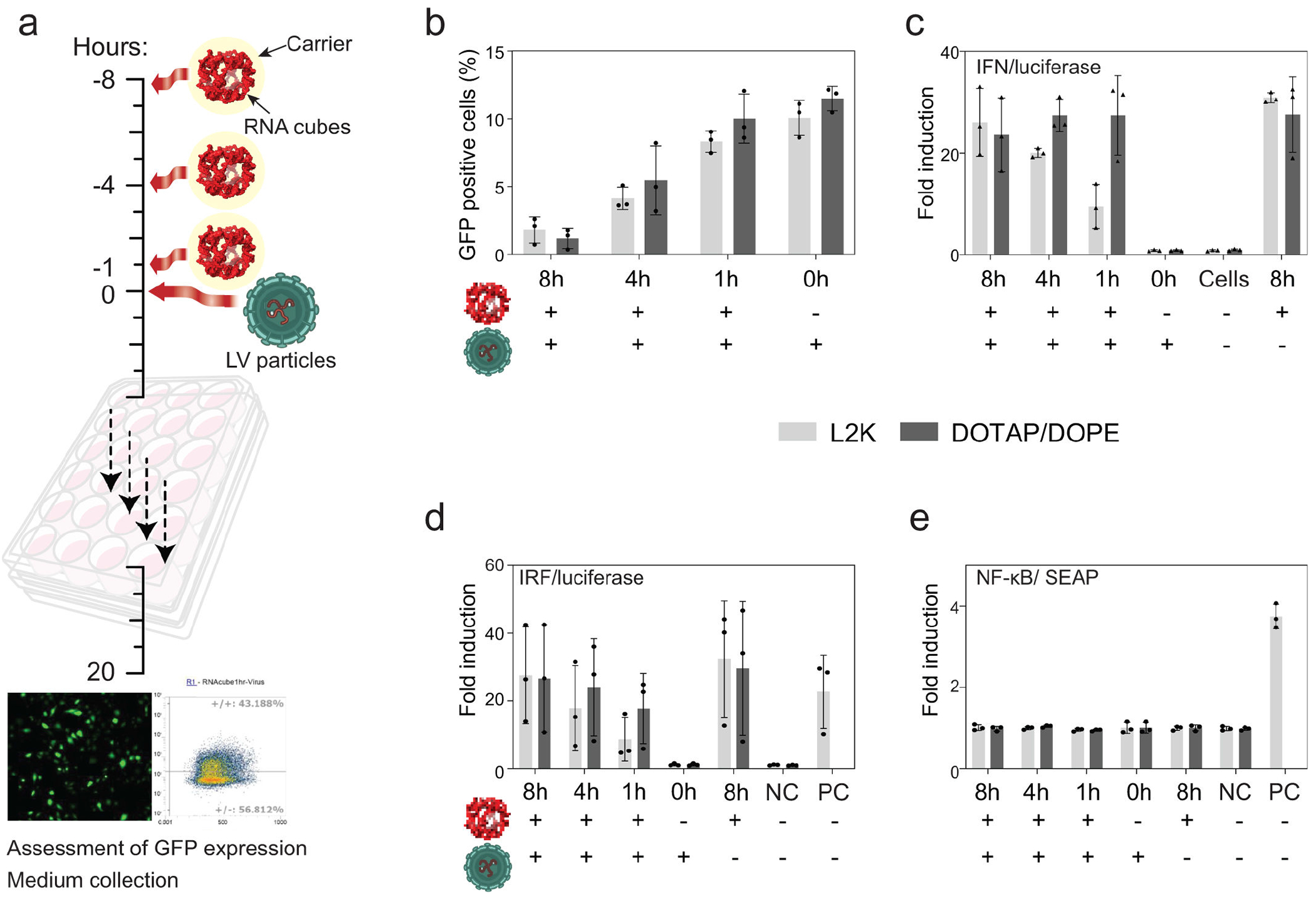
RNA cube iNANPs inhibit transduction of A549 cells by LV particles. (a) Schematic of experimental design. (b) Delivery of iNANPs to A549 cells induces a time-dependent antiviral state, independent of carrier complexation. (c) IFN release into the medium over time, measured by luciferase induction in HEK-Null cells after supplementing with conditioned media from A549 cells treated with RNA cubes harvested 20 h post-infection. (d) A similar time-dependent response was observed in THP-1 dual cells through the IRF pathway. (e) No activation was detected through the NF-κB pathway. Data are presented as mean ± SD, *n* = 3. PC, positive control: 2 μg/mL 2′3′-cGAMP (IRF/Luciferase) and 3 μg/mL Pam3CSK4 (NF-κB/SEAP).

To investigate how delivery method and exposure time affect antiviral activity, we transfected A549 cells with our representative iNANPs delivered using either L2K or DOTAP/DOPE and exposed the cells for 8, 4, or 1 h before replacing the medium with fresh medium containing lentiviral particles (Fig. 3a). We used LV particles to study iNANP-mediated antiviral signaling because they efficiently deliver reporter transgene into target cells while mimicking viral infection, thereby providing a safe and controllable system to evaluate how iNANPs enhance innate immune responses, including interferon production. Additionally, LV-based reporter systems allow a quantitative, real-time monitoring of antiviral pathway activation in response to treatments. The efficiency of LV infection-mediated transduction was evaluated after 20 h by microscopy and flow cytometry for percentage of GFP-positive cells. The GFP expression in A549 cell population reflected success of infection or, inversely, induction of antiviral state.

The length of cell exposure to iNANPs prior to LV-mediated transduction affected the percentage of LV coding GFP transgene integrated to host A549 cells (Fig. 3b). The similar percentage of infected cells suggests that transfection agents did not play a role in the induction of antiviral state by delivery of RNA cubes. As production of IFNs is a hallmark of innate immune system activation, we treated HEK-Lucia Null cells with conditioned media harvested from transfected A549 cells 20 h after addition of LV particles (Fig. 3c). Transcription of luciferase is induced by IFN present in media and, after its expression, is secreted into the medium. Interestingly, the level of luciferase production induced by IFNs in medium from A549 cells with RNA cube iNANPs delivered by DOTAP/DOPE just for 1 h was similar to 4- and 8-h treatment, suggesting a rapid release of transfected cargo with quick recognition by cells. In comparison, luciferase production in cells transfected by iNANPs complexed with L2K is time dependent, suggesting their gradual release (Fig. 3c).

Next, we examined if conditioned medium containing IFNs and other cytokines activate IRF pathway and/or pathways leading to NF-κB inflammation pathway in THP1-Dual reporter cells. We detected similar trends of luciferase expression under IRF-controlled pathways, as in HEK Null cells (Fig. 3d), and no SEAP expression in THP1-Dual cells was observed (Fig. 3e), suggesting that cytokines in conditioned media only stimulate pathways merging to IRF signaling (Fig. 3d). We evaluated the viability of A549 cells and reporter cell lines after 20 h post-infection (h p.i.) or 24 h post media treatment, and no significant decrease in viability was observed (not shown).

**FIG 4:**
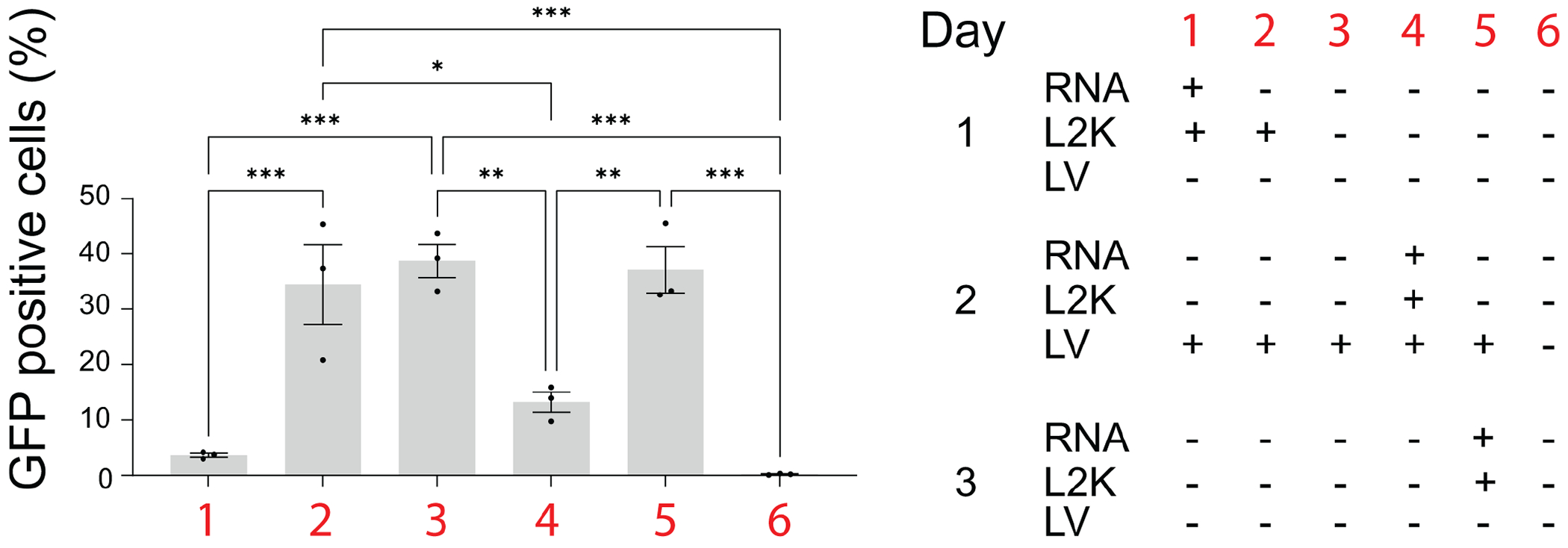
Percentage of GFP-positive cells after transduction of HEK293-FT cells with LV particles produced under various conditions (1 to 6) as specified in the table on right side, *N* = 3, ±SEM. RNA-RNA cubes with six 5′-triphosphate groups. *P < 0.05; **P < 0.01; ***P < 0.001.

Next, we asked whether treatment of LV producer cells could inhibit or reduce LV particle production. To test this, we used HEK293-FT cells, which were transfected with plasmids necessary for LV production. We observed a lower number of GFP-positive HEK293-FT cells after transduction with media harvested from LV producing cells transfected with RNA cubes prior to delivery of plasmids coding LV genes. Similarly, co-transfection of RNA cubes and plasmids reduced LV production. Delivery of iNANPs after transfection with LV plasmids did not affect LV particle production (Fig. 4).

### iNANP-induced antiviral response inhibits replication of different viruses

**FIG 5:**
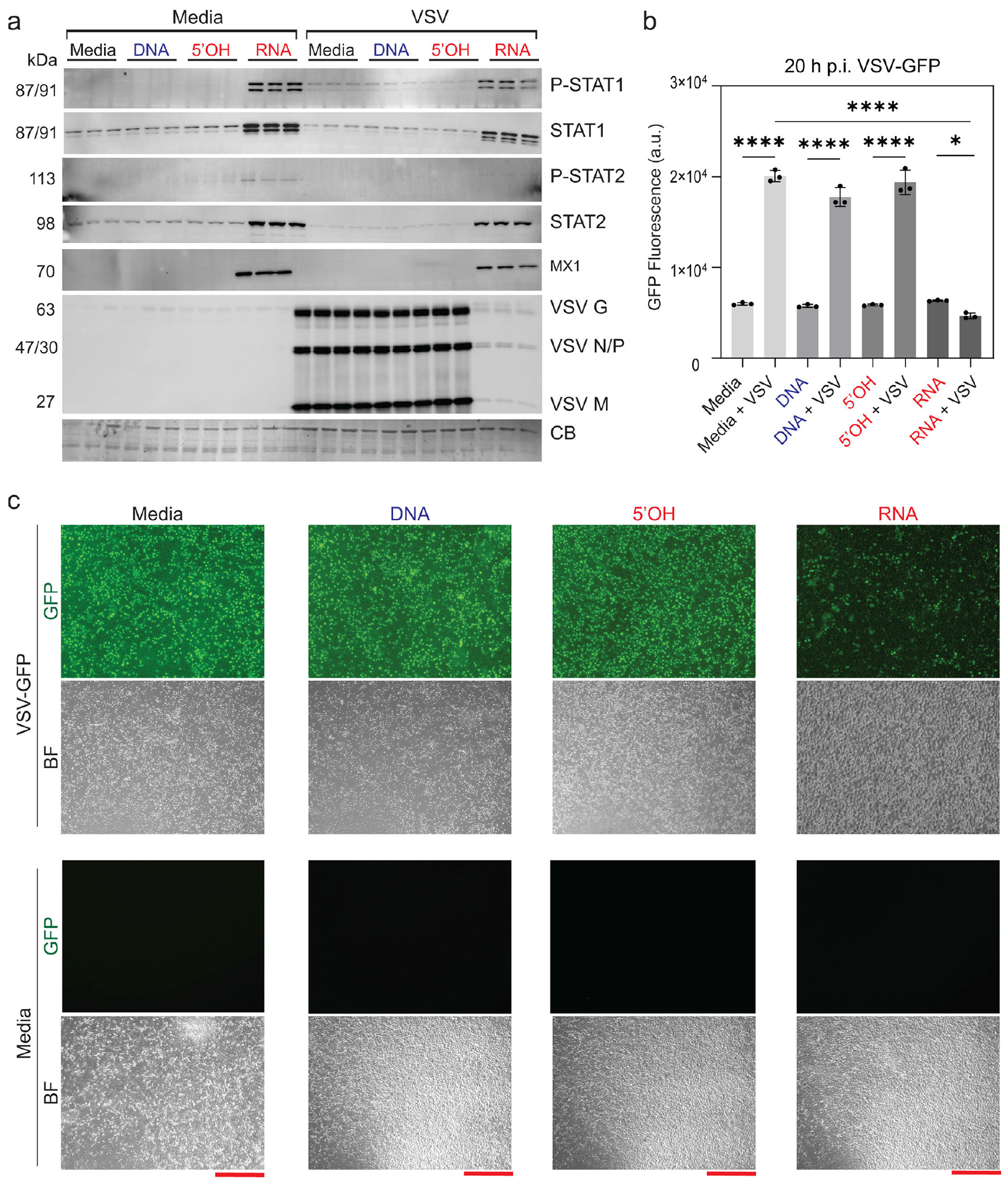
RNA cube iNANPs effectively block VSV replication and induce antiviral activity in A549 cells. Cells were transfected with iNANP variants RNA (RNA cubes with six 5′-triphosphate groups), 5′OH (RNA cubes with six hydroxyl groups at the 5′ ends), DNA (DNA cubes), or media alone for 8 h, followed by infection with VSV-GFP at an MOI of 0.1 (BHK-21 titer). At 20 h p.i.: (a) protein samples were analyzed by western blotting, with equal loading verified by Coomassie blue (CB) staining. Brightness and contrast were adjusted uniformly across the entire image to improve band visualization without selective enhancement, splicing, or alteration of the original data. (b) GFP fluorescence was measured before protein collection. (c) Fluorescence microscopy images were acquired to assess VSV-driven GFP expression (BF, bright field; GFP, green fluorescent protein; scale bars = 40 μm). Data are presented as mean ± SD, *n* = 3. Statistical significance was determined by one-way ANOVA with Fisher’s least significant difference test, comparing each virus condition to virus + RNA and media alone to virus + RNA. ns, not significant; *P < 0.05; ****P < 0.0001.

*In vitro* infection of cells with replication-competent viruses reflects all stages of the viral life cycle. Therefore, we employed several replication-competent viruses that express GFP transgene as a marker of successful infection/replication to describe the efficiency of iNANP-primed cell infection. VSV is commonly used to examine various aspects of virus-host interactions, including activation of innate immune responses. To examine the effect of different cubes on VSV replication, we transfected A549 cells with either DNA, 5′OH-RNA, or RNA cubes (or media only). Eight hours later, cells were infected with VSV at a multiplicity of infection (MOI) of 0.1. 20 h p.i., and accumulation of VSV-encoded proteins was evaluated by western blot analysis, microscopy, and GFP fluorescence quantification. RNA cube-transfected cells exhibited markedly reduced levels of VSV protein accumulation compared to all control conditions (Fig. 5), along with a reduction in VSV-encoded GFP fluorescence, as measured by both plate reader and microscopy (Fig. 5b and c). Importantly, cells treated with RNA cube iNANPs displayed elevated levels of phosphorylated STAT1 and STAT2 and total STAT1 and STAT2. Furthermore, the interferon-stimulated gene MX1, which is known to have wide antiviral activity against various RNA and DNA viruses, has been upregulated, even in the absence of viral infection, too (Fig. 5a). These data suggest that RNA cubes alone are sufficient to induce a robust IFN I response, establishing an antiviral state that impairs VSV replication.

To examine if RNA cube-mediated inhibition of VSV replication in A549 cells correlated with increased secretion of IFNs, A549 cells were transfected with either three iNANP variants or just media for 8 h, and then infected with VSV at an MOI of 0.1. At 20 h p.i., the cell culture medium was collected and examined for IFN production using the Human Interferon 9-Plex Discovery Assay Array.

**FIG 6:**
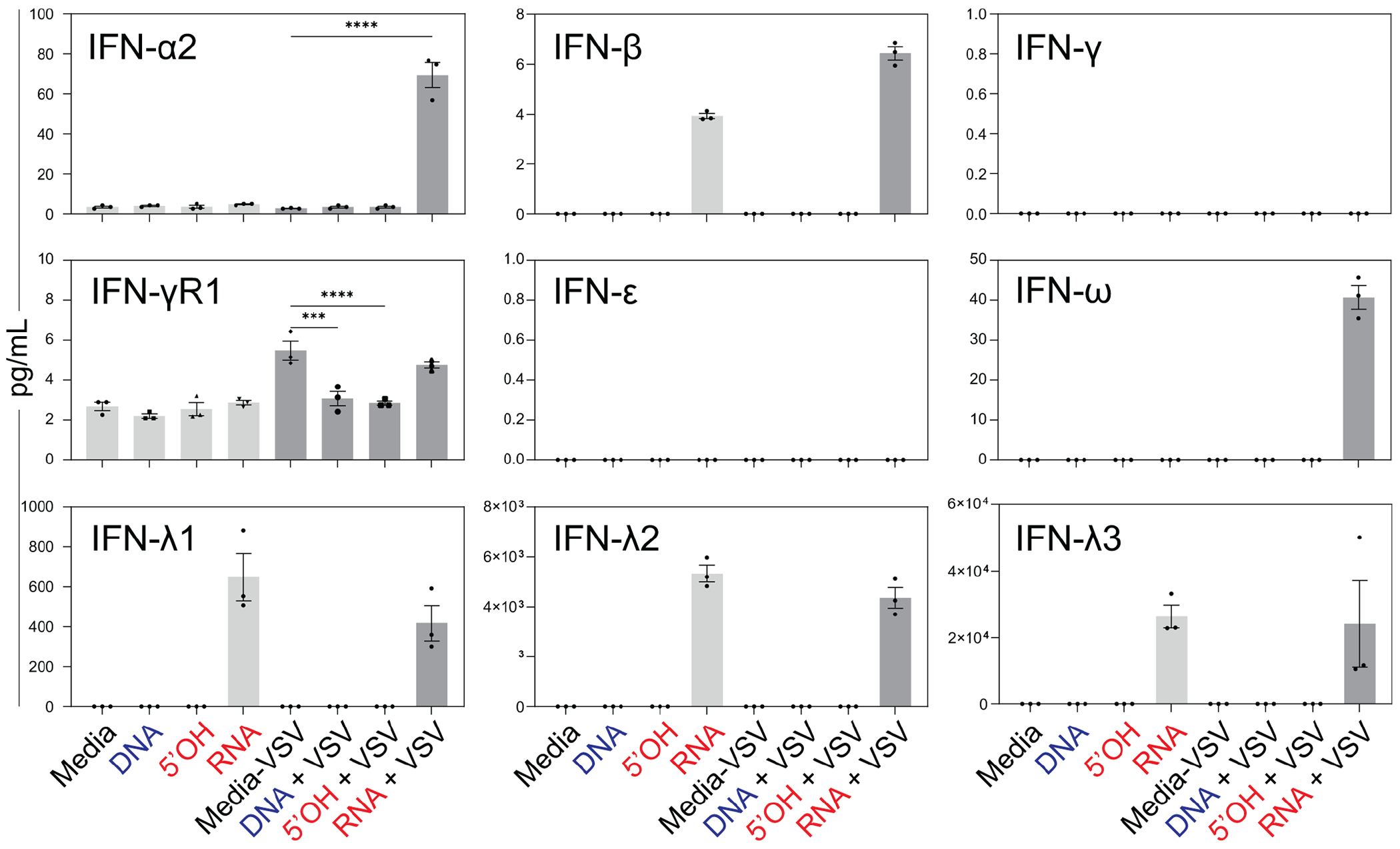
Effect of iNANPs on secretion of different IFNs by A549 cells. Cells were transfected with iNANP variants RNA (RNA cubes with six 5′-triphosphate groups), 5′OH (RNA cubes with six hydroxyl groups at the 5′ ends), DNA (DNA cubes), or media alone for 8 h, followed by infection with VSV-GFP at an MOI of 0.1 (BHK-21 titer). At 20 h p.i., culture supernatants were collected and analyzed using the Human Interferon 9-Plex Discovery Assay Array (HDIFN9, Eve Technologies). Data are shown as mean ± SEM. Statistical significance was assessed by Student’s *t*-test with a cut-off of 1,000 ng/mL applied across all cytokines. ns, not significant; *P < 0.05; ***P < 0.001; ****P < 0.0001.

As shown in Fig. 6, only RNA cube iNANPs (but not other variants) stimulated the secretion of IFN-β and IFN-λ 1, IFN-λ-2, or IFN-λ-3, independent of VSV infection. Following VSV infection, increased secretion of IFN-α2 and IFN-ω was observed only in cells transfected with RNA cubes (in addition to the secretion of IFN-β and IFN-λ1-3). IFN-γ and IFN-ε both remained under detectable levels under all conditions. In contrast, IFN-γR1 levels remained between 2 and 6 pg/mL across all conditions. Interestingly, the induction of IFN-α2 and IFN-ω was observed only with the addition of VSV infection in the RNA cube condition.

**FIG 7:**
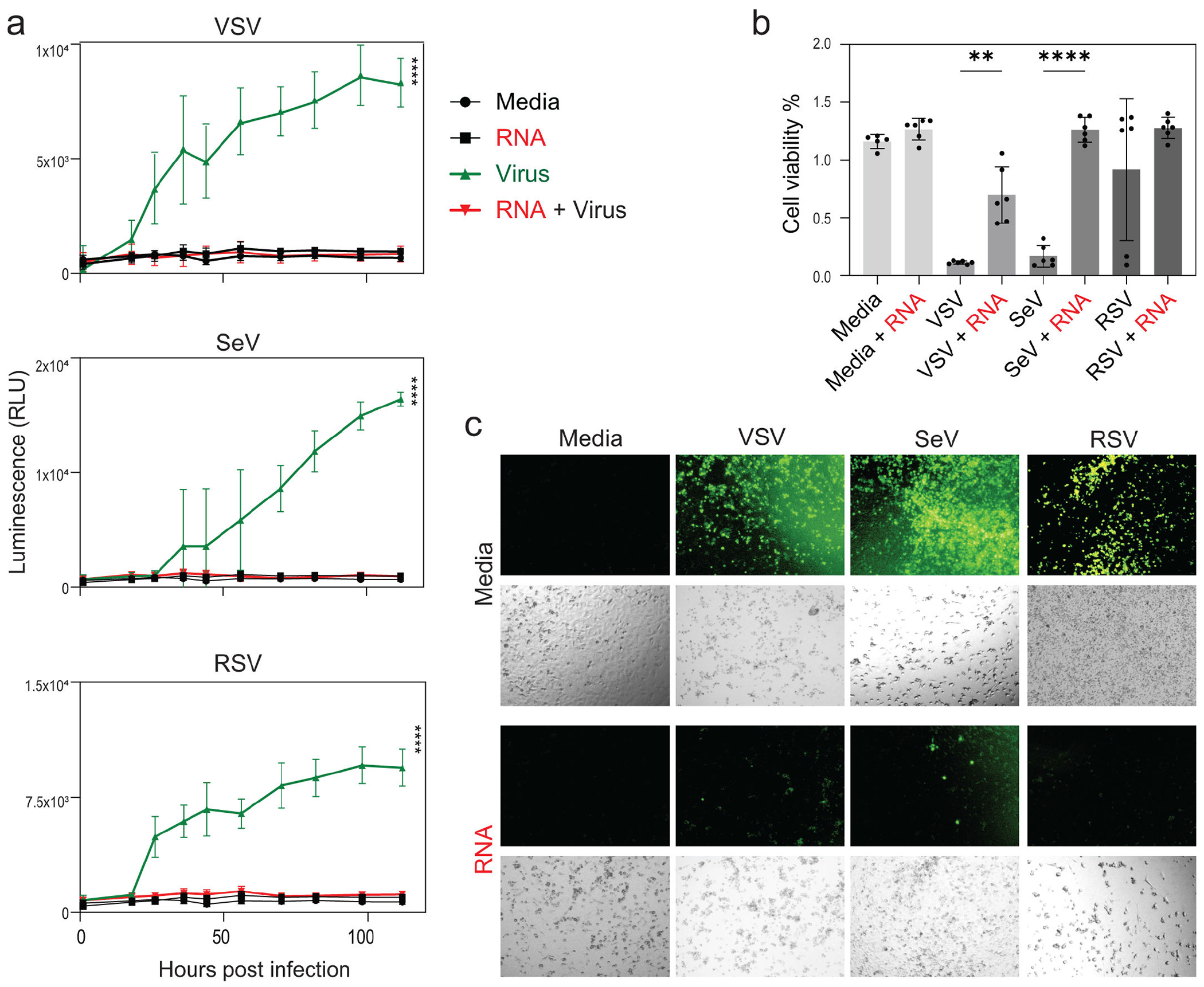
Effect of RNA cube iNANPs on VSV-GFP, RSV-GFP, and SeV-GFP. (a) A549 cells were transfected with RNA cubes (with six 5′-triphosphate groups) for 8 h then infected with either VSV-GFP, RSV-GFP, or SeV-GFP (or mock-infected) with an MOI of 0.1. GFP fluorescence was measured between 1 and 114 h p.i. Results were analyzed to determine significance using the Student’s *t*-test comparing 114 h p.i. of “Virus” compared to “RNA cubes + Virus”. ***P < 0.001. (b) Cell viability was measured at 118 h p.i. using a WST-8 cell viability assay. Each data point was divided by the average mock value to obtain the percentage viability to mock. The data points and error bars shown represent the means and standard deviation of the means, respectively. Results were analyzed to determine significance using a one-way ANOVA with Fisher’s least significant difference test between each virus and virus + RNA cube in addition to media alone and each virus + RNA cube. ns, not significant, **P < 0.01, ****P < 0.0001. (c) Fluorescence microscopy images were acquired to assess GFP expression in infected cells.

To evaluate whether the antiviral activity of RNA cubes extends beyond VSV to other clinically relevant RNA viruses, we next examined their effects on SeV and RSV, both of which are known to target the respiratory epithelium and employ diverse immune evasion strategies. A549 cells were transfected with RNA cubes or left untreated, followed by infection 8 h later with VSV, SeV, or RSV at an MOI of 0.1. Viral replication kinetics were monitored by measuring GFP fluorescence over 120 h p.i. (Fig. 7a). Across all viral infections, RNA cube iNANP pre-treatment consistently suppressed virus-encoded GFP fluorescence, indicating impaired viral replication. Notably, while mock-treated cells supported robust replication of each virus, iNANP-transfected cells exhibited significantly lower fluorescence levels throughout the time course. Correspondingly, cell viability at 120 h p.i. was higher in the RNA cube-treated, virus-infected groups compared to virus-only controls (Fig. 7b), suggesting that RNA cube iNANP-mediated antiviral responses not only suppress viral replication but also confer protection against virus-induced cytopathic effects. These findings support the broad-spectrum antiviral potential of iNANPs and their utility in protecting respiratory epithelial cells from diverse RNA virus infections.

### RNA cubes induce a conserved antiviral state across multiple human cell lines

**FIG 8:**
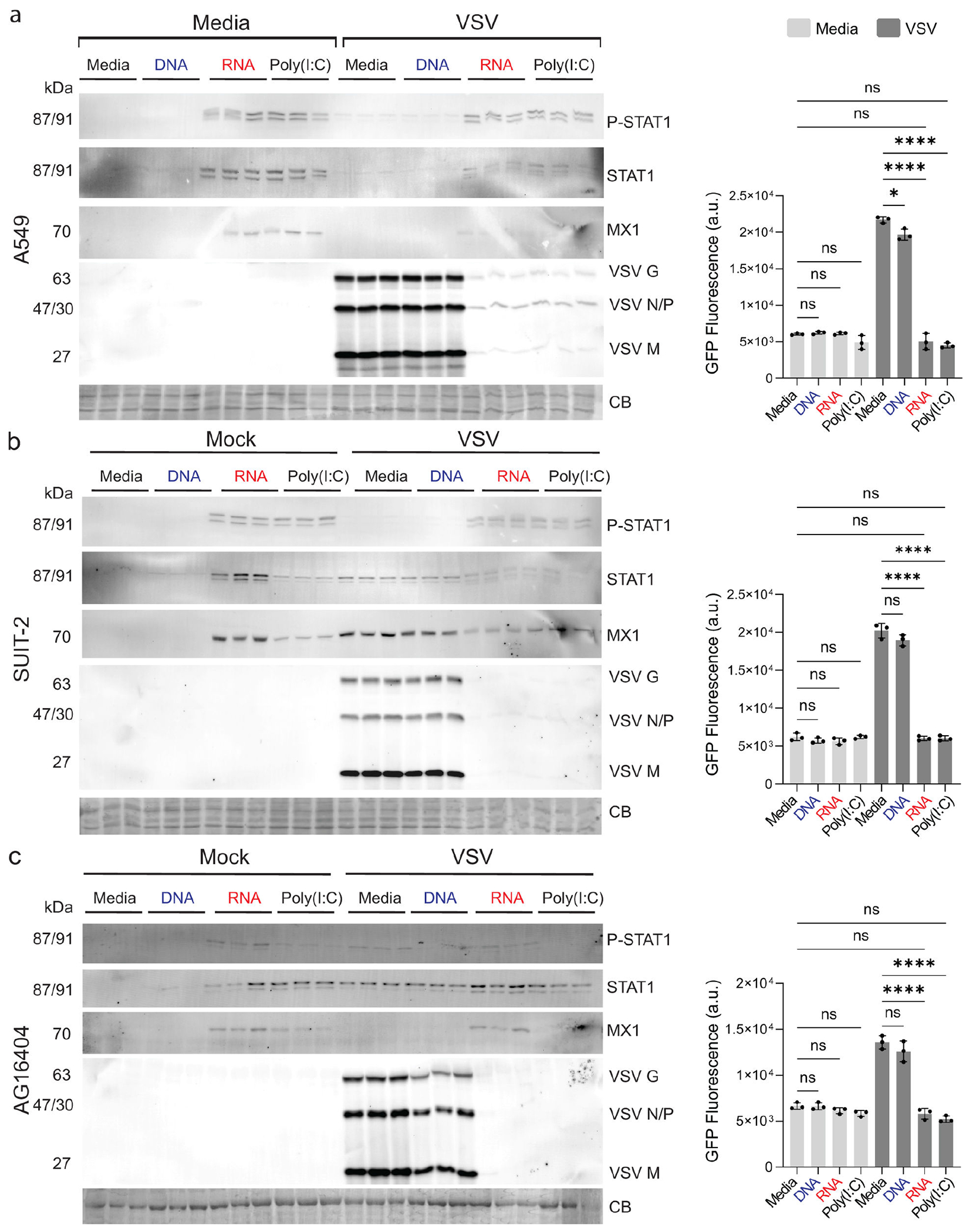
Effect of iNANPs and poly(I:C) on antiviral activity across multiple human cell lines. (a) A549, (b) SUIT-2, or (c) AG16404 cells were transfected with either RNA cubes (with six 5′-triphosphate groups), DNA cubes, poly(I:C), or media alone for 8 h, followed by infection with VSV-GFP at an MOI of 0.1 (BHK-21 titer). At 20 h p.i., protein samples were analyzed by western blotting, with equal loading verified by Coomassie blue (CB) staining. Brightness and contrast were adjusted uniformly across the entire image to improve band visualization without selective enhancement, splicing, or alteration of the original data. GFP fluorescence was measured before protein collection (right panel). Data are presented as mean ± SD, *n* = 3. Statistical significance was determined by one-way ANOVA with Fisher’s least significant difference test, comparing each virus condition to virus + RNA and media alone to virus + RNA. ns, not significant; *P < 0.05; ****P < 0.0001.

To evaluate the consistency of RNA cube-induced antiviral responses, we examined their activity across three human cell lines representing distinct tissue origins: A549, SUIT-2, and AG16404. Cells were transfected with media, RNA cubes, DNA cubes, poly(I:C), or media alone for 8 h prior to infection with VSV-GFP at MOI 0.1 (BKH-21 titer). Viral replication and host antiviral signaling were assessed 20 h p.i. by monitoring VSV-driven GFP expression and by western blot analysis of key antiviral markers (Fig. 8).

Across all three cell lines, RNA cube treatment consistently resulted in robust activation of the type I interferon signaling pathway, as evidenced by increased phosphorylation of STAT1 and induction of the interferon-stimulated gene MX1. This activation was accompanied by a marked reduction in VSV replication demonstrated by decreased levels of VSV proteins on western blots and reduced GFP fluorescence relative to mock- or DNA cube-treated controls. In contrast, DNA cubes did not induce detectable STAT1 phosphorylation or MX1 expression and failed to inhibit VSV replication, confirming that the antiviral effects were RNA-dependent rather than a consequence of nanoparticle structure alone.

Notably, the antiviral effects observed with RNA cube iNANPs were comparable to those induced by poly(I:C), a well-established interferon-stimulating dsRNA control, although the magnitude of response varied modestly between cell lines. For example, while differences between RNA cube- and poly(I:C)-transfected A549 are negligible (Fig. 8a), RNA cubes transfected in SUIT-2 appeared to result in increased levels of STAT1 and MX1 when compared to poly(I:C) treatments (Fig. 8b). Consistently, RNA cube-transfected AG16404 resulted in more robust STAT1 activation compared to poly(I:C) (Fig. 8c). Together, these data demonstrate that RNA cubes reliably activate STAT1-mediated antiviral signaling and suppress viral replication across diverse human cell types, supporting their ability to induce a conserved antiviral state.

**FIG 9:**
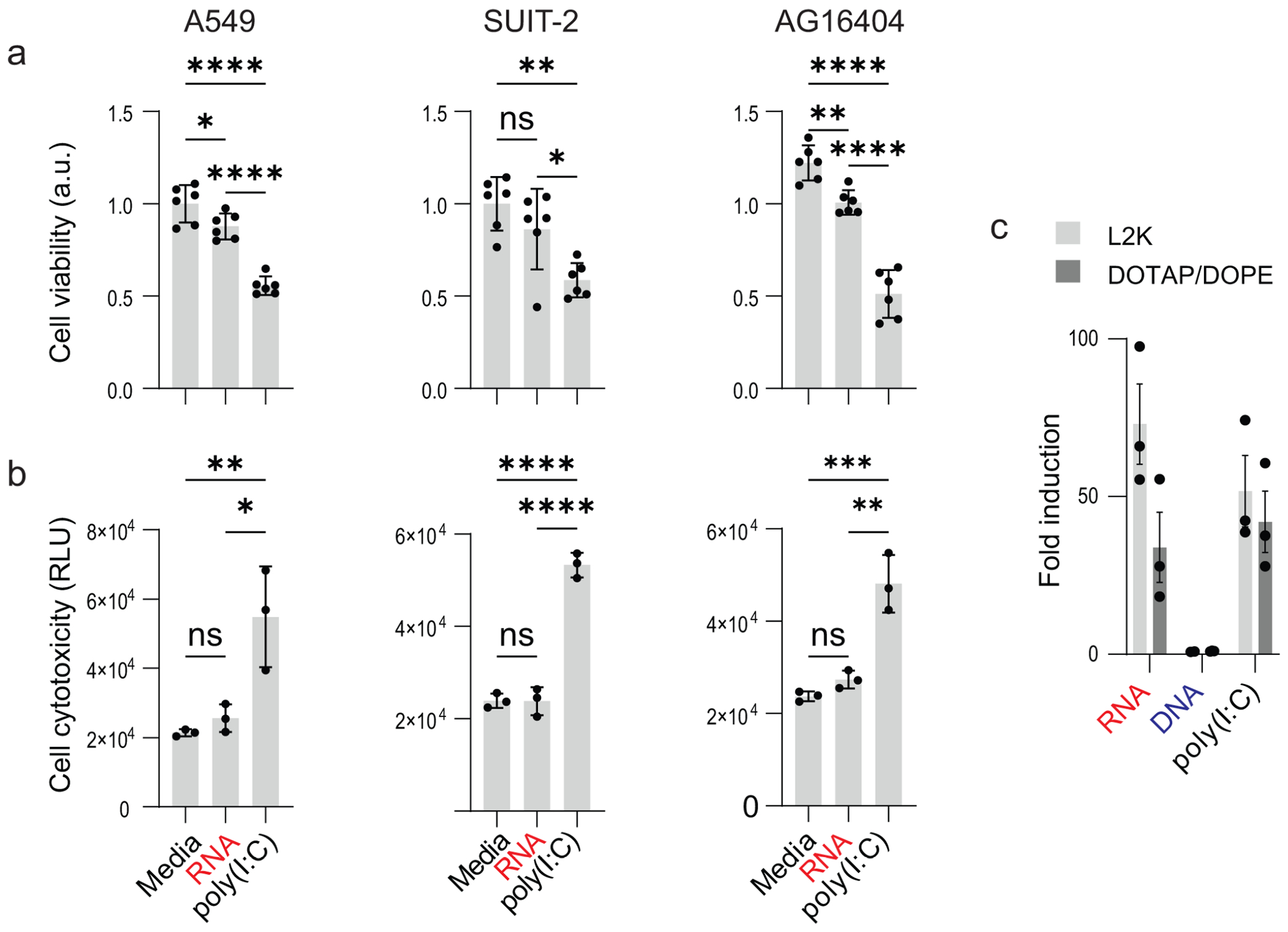
Effect of RNA cube iNANPs and poly(I:C) on cell viability and cytotoxicity. A549, SUIT-2, or AG16404 cells were transfected with either RNA (RNA cubes with a 5′-triphosphate group), poly(I:C), or media alone for 28 h before (a) cell viability and (b) cell cytotoxicity were measured. Data are presented as mean ± SD, *n* = 3 (cell cytotoxicity), and *N* = 6 (cell viability). Statistical significance was determined by one-way ANOVA with Fisher’s least significant difference test, comparing each virus condition to virus + RNA and media alone to virus + RNA. ns, not significant; *P < 0.05; **P < 0.01; ***P < 0.001; ****P < 0.0001. (c) Production of luciferase in HEK-Lucia-RIG-I cells after induction of RIG-I by 5 nM RNA cubes, DNA cubes, and 1 μg/mL of poly(I:C), *n* = 3, ±SEM.

To determine whether the antiviral activity of RNA cubes was associated with cellular toxicity, we assessed cell viability and cytotoxicity in A549, SUIT-2, and AG16404 cells 28 h after transfection with RNA cubes or poly(I:C) (Fig. 9). Media-treated cells served as a baseline control. This analysis was performed to directly compare the cellular tolerability of RNA cube with that of poly(I:C), a potent but often cytotoxic interferon-inducing agent.

Across all three cell lines, RNA cube treatment resulted in minimal reductions in cell viability (Fig. 9a) and low levels of cytotoxicity (Fig. 9b) comparable to media-treated controls. In contrast, poly(I:C) treatment led to a pronounced decrease in cell viability (Fig. 9a) accompanied by significantly elevated cytotoxicity signals (Fig. 9b). This effect was consistent in all tested cell types, indicating that poly(I:C) induced substantial cellular stress or death under conditions where RNA cubes remained well tolerated. However, expression of luciferase in reporter cell line suggests similarly effective activation of RIG-I by RNA cubes and poly(I:C) (Fig. 9c).

## DISCUSSION

This study demonstrates that iNANPs rapidly activate innate immune pathways to induce a potent antiviral state in respiratory epithelial cells. We show that our formulations robustly trigger type I and III interferon responses through RIG-I and TLR7 signaling, with a particularly strong induction of IFN-β and IFN-λ1, IFN-λ2, and IFN-λ3 prior to the viral challenge. Both type I and III IFNs play crucial roles in the formation of an antiviral state in which cells are primed to resist viral infection. Secretion of IFNs subsequently boosts cell defense in an autocrine manner and alerts uninfected, neighboring cells in a paracrine manner. Importantly, this response was independent of the delivery vehicle but highly dependent on the timing of exposure, highlighting the rapid recognition and action of iNANPs. Pre-treatment of A549 cells with formulations effectively inhibited replication of multiple clinically relevant respiratory RNA viruses, including VSV, RSV, and SeV, while also protecting cells from virus-induced cytopathic effects. Binding of IFNs to interferon receptors triggers signaling pathways that lead to the expression of ISGs that create a hostile environment for the virus, decreasing its ability to replicate (36). Mechanistically, we found that iNANP-mediated induction of ISGs, such as STAT1, STAT2, and MX1, was sufficient to suppress viral protein synthesis and spread. These findings establish iNANPs as a promising broad-spectrum antiviral platform with prophylactic potential, particularly in high-risk settings where viral exposure is probable, but the causative pathogen is unknown. Future studies should focus on optimizing carrier formulations and *in vivo* delivery strategies to advance the translation of iNANPs into practical antiviral interventions for respiratory infections. Additionally, incorporating antiviral siRNAs and other therapeutic nucleic acids into the iNANP structure may provide synergistic effects in antiviral therapy and should be investigated.

## MATERIALS AND METHODS

### Synthesis of iNANPs

RNA cube monomers with 5′-triphosphate groups were synthesized in laboratory by *in vitro* run-off transcription, while 5′OH RNA and DNA monomers were purchased from Integrated DNA Technologies (IDT). RNA cubes with 5′PPP: rA: 5′-PPP-rGrGrCrArArCrUrUrU rGrArUrCrCrCrUrCrGrGrUrUrUrArGrCrGrCrCrGrGrCrCrUrUrUrUrCrUrCrCrCrArCrArCrUrUrrCrArCrG −3′, rB: 5′-PPP-rGrGrGrArArArUrUrUrCrGrUrGrGrUrArGrGrUrUrUrUrGrUrUrGrC rCrCrGrUrGrUrUrUrCrUrArCrGrArUrUrArCrUrUrUrGrGrUrC -3′, rC: 5′-PPP-rGrGrArCrArUrUrUrUrCrGrArGrArCrArGrCrArUrUrUrUrUrUrCrCrCrGrArCrCrUrUr UrGrCrGrGrArUrUrGrUrArUrUrUrUrArGrG -3′, rD: 5′-PPP-rGrGrC rGrCrU rUrUrU rGrArC rCrUrU rCrUrG rCrUrU rUrArUrGrUrCrCrCrCrUrArUrUrUrCrUrUrArArUrGrArCrUrUrUrUrGrG rCrC-3′, rE: 5′-PPP-rGrGrG rArGrArUrUrUrArGrUrCrArUrUrArArGrUrUrUrUrArCrArArUrCrCr GrCrUrUrUrGrUrArArUrCrG rUrArGrUrUrUrGrUrGrU-3′, rF: 5′-PPP-rGrGrGrArUrCrUrUrUrAr CrCrUrArCrCrArCrGrUrU rUrUrGrCrUrGrUrCrUrCrGrUrUrUrGrCrArGrArArGrGrUrCrUrUrUr CrCrGrA-3′. RNA cubes with 5′OH: rA: 5′-OH-rGrGrCrArArCrUrUrUrGrArUrCrCrCrUrCrGrGrUrUrUrArGrCrGrC rCrGrGrCrCrUrUrUrUrCrUrCrCrCrArCrArCrUrUrUrCrArCrG −3′, rB: 5′-O H-rGrGrGrArArA rUrUrU rCrGrUrGrGrUrArGrGrUrUrUrUrGrUrUrGrCrCrCrGrUrGrUrUrUrCr UrArCrGrArU rUrArCrUrUrUrGrGrUrC-3′, rC: 5′-OH-rGrGrArCrArUrUrUrUrCrGrArGrArCr ArGrCrArUrUrUrUrUrUrCrCrCrGrArCrCrUrUrUrGrCrGrGrArUrUrGrUrArUrUrUrUrArGrG-3′, rD: 5′-OH-rGrGrCrGrCrUrUrUrUrGrArCrCrUrUrCrUrGrCrUrUrUrArUrGrUrCrCrCrCrUrArUr UrUrCrUrUrArArUrGrArCrUrUrUrUrGrGrCrC-3′, rE: 5′-OH-rGrGrGrArGrArUrUrUrArGrUr CrArUrUrArArGrUrUrUrUrArCrArArUrCrCrGrCrUrUrUrGrUrArArUrCrGrUrArGrUrUrUrGrUrGrU-3′, rF: 5′-OH-rGrGrGrArUrCrUrUrUrArCrCrUrArCrCrArCrGrUrU rUrUrGrCrUrGrUrCrUrCrGrUrUrUrG rCrArGrArArGrGrUrCrUrUrUrCrCrGrA -3′. DNA cubes: dA: 5′-OH-GGCA ACTTTGATCCC TCGGTTTAGCGCCGGCCTTTTCTCCCACACTTTCACG-3′, dB: 5′-OH-GGGAAA TTTCGT GGTAGGTTTTGTTGCCCGTGTTTCTACGATTACTTTGGTC-3′, dC: 5′-OH-GGACATTTT CGAGACAGCATTTTTTCCCGACCTTTGCGGATTGTATTTTAGG-3′, dD: 5′-OH-GGCGCT TTTGA CCTTCTGCTTTATGTCCCCTATTTCTTAATGACTTTTGGCC-3′, dE: 5′-OH-GGG AGATTTAGTCA TTAAGTTTTACAATCCGCTTTGTAATCGTAGTTTGTGT-3′, dF: 5′-OH-GGGATCTTTACCTACCAC GTTTTGCTGTCTCGTTTGCAGAAGGTCTTCCGA-3′. Run-off transcription was performed on PCR-amplified DNA. For transcription, a solution of 80 mM HEPES-KOH (pH 7.5), 2.5 mM spermidine, 50 mM DTT, 2 mM MgCl2, 25 mM rNTPs, 0.2 μM DNA templates, and ~100 units/μL of T7 RNA polymerase (purified in house) was incubated for 4–16 h at 37°C. DNA template in transcription mix was degraded by RQ DNase I (Promega) for 30 min at 37°C. Crude mixture was purified on 8% denaturing polyacrylamide electrophoresis.

### Assembly of iNANPs

All iNANPs were produced by mixing six cognate strands at equimolar ratio in a one-pot assembly. Initial thermal denaturation for 2 min at 95°C was followed by incubation for 2 min at 45°C, with the assembly buffer added to reach a final concentration of 89 mM tris-borate, 2 mM MgCl_2_, and 50 mM KCl. The samples were then incubated for an additional 20 min at 45°C. Samples were stored at 4°C indefinitely. To confirm assemblies, samples were assessed with Native-PAGE, 8% 37.5:1 acrylamide/bis-acrylamide, with total ethidium bromide staining and visualized using ChemiDoc.

### Atomic force microscopy

Next, 5 μL (50 nM) of each iNANP was deposited on APS-modified mica, incubated for ~ min, and air-dried, as described previously according to established protocols (22). Briefly, AFM was performed using the MultiMode AFM Nanoscope IV System (Bruker Instruments, Santa Barbara, CA) in tapping mode. The images were recorded with a 1.5 Hz scanning rate using a TESPA-300 probe from Bruker with a resonance frequency of 320 kHz and a spring constant of approximately 40 N/m. Images were processed by the FemtoScan online software package (Advanced Technologies Center, Moscow, Russia).

### Transmission electron microscopy

After complexation, 5 μL of each sample was dropped onto a carbon-coated 400 mesh Cu/Rh grid (Ted Pella, Redding, CA, USA) and stained with 5 μL of 1% uranyl acetate (Polysciences, Warrington, PA, USA), which was prepared in filtered distilled water. A FEI Talos L120C TEM with a Gatan 4 k × 4 k OneView camera was used to image the grids.

### Lentiviral vector production

One day prior to transfection, 6 × 10^6^ HEK293FT cells were seeded in the 150 cm^2^ tissue culture dish. To produce lentiviral vector particles in one dish, 7.65 μg of pNL(CMV)EGFP/CMV/WPREΔU3 vector plasmid derived from the pNL-EGFP/CMV/WPREDU3 plasmid (37), 5.1 μg pCD/NL-BH*ΔΔΔ packaging helper plasmid (38), and 1.95 μg of pCEF-VSV-G envelope (39) were transfected using PEI. The next day, the medium was replaced with fresh DMEM medium, 100 U/mL penicillin, and 100 μg/mL streptomycin. After 72 h post-transfection, medium was aspirated and spinned for 5 min at 500 × *g* at 4°C. Supernatant was subsequently filtered through 0.45 μm filter and mixed with Lenti-X lentiviral concentrator (Clontech, Mountain View, CA, USA) in a 3:1 ratio. After 4 h of incubation at 4°C, vector containing medium was centrifuged for 2 h at 3,000 × *g* for 45 min at 4°C. Pellet was 10-fold concentrated in PBS, aliquoted, and stored in −80°C.

### A549 transfection and LV transduction

Before treatment, A549 cells were plated in a 24-well plate at 80,000 cells/well density. Transfection of A549 with 50 nM RNA cube (or other iNANPs) was carried out using Lipofectamine 2000 or DOTAP/DOPE as a carrier for the final volume of 500 μL. Cells were transfected −8, −4, and −1 h before media were removed and replaced with 500 μL DMEM per well supplemented with lentiviral vector and 8 μg/mL of polybrene. Cells were subsequently incubated for an additional 20 h. Afterward, GFP-expressing cells were evaluated by fluorescent microscopy and harvested for flow cytometry.

### IFN evaluation by reporter cell lines

The reporter cell lines here were used in the study. The reporter is expressed as follows: THP1-Dual cells, a human monocyte line, express luciferase under the control of an ISG54 minimal promoter coupled with five IFN-stimulated response elements (ISRE). Activation of RIG-I in HEK-Lucia RIG-I cells induces expression of the Lucia luciferase reporter under an IFN-inducible ISG54 promoter enhanced by multimeric ISRE. In HEK-Blue hTLR7 reporter cells, TLR7 activation drives expression of an NF-κB/AP-1-inducible secreted embryonic alkaline phosphatase (SEAP) reporter. Both HEK-Lucia RIG-I and HEK-Blue hTLR7 cells were seeded 24 h prior to transfection at a density of 40,000 cells per well in a 96-well plate. The level of Luciferase and SEAP was assayed by addition of corresponding substrate after 24 h. The HEK-Lucia Null cells are HEK293-derived cells that express the Lucia luciferase protein under the control of the IFN-inducible promoter. For the presence of secreted IFNs in medium as a response to the RNA cube-induced antiviral state, HEK-Null cells were plated at 40,000 cells per well, while THP1-Dual cells at 100,000 cells per well in a 96-well plate. After 24 h, conditioned media were transferred from A549 treated with iNANPs with or without virus. Cells were incubated for additional 16–20 h. Cell viability, secretion of luciferase, or secreted embryonic alkaline phosphatase (SEAP) was measured with the following assays CellTiter 96 AQueous One Solution Cell Proliferation Assay (Promega), QUANTI-Luc (Invivogen), or QUANTI-Blue (Invivogen), respectively.

### Flow cytometry

Media were removed, and cells were washed with PBS. After trypsinization, cells were centrifuged at 500 × *g* at 4°C for 5 min. After removal of supernatant, cells were resuspended in 1× PBS supplemented with 1% BSA, 0.2 mM EDTA, and SYTOX red. After 15 min of incubation at room temperature, cells were finally analyzed on an Attune Nxt flow cytometer.

### Western blot

A549 cells were plated in a 24-well plate at 80,000 cells/well density in 450 μL per well. Transfection of A549 with 50 nM of iNANPs was carried out using DOTAP as a carrier for the final volume of 500 μL. Virus was then added at MOI of 0.1 in serum-free DMEM medium and incubated for 1 h at 37°C. After 1 h of incubation, the medium was removed, and 5% FBS-containing medium was added to the cells. Cells were then lysed, and total protein was isolated 24 h p.i. using a buffer containing 1 M Tris-HCl (pH 6.8), 10% glycerol, 2% SDS, 5% beta-mercaptoethanol, and 0.02% (wt/vol) bromophenol blue. Total protein was separated by electrophoresis on 10% or 15% SDS-PAGE gels and electroblotted onto polyvinyl difluoride (PVDF) membranes. Membranes were blocked by using 5% nonfat powdered milk or BSA in TBS-T (0.5 M NaCl, 20 mM Tris [pH 7.5], 0.1% Tween 20) for 1 h at room temperature. Membranes were then incubated in TBS-T with 5% BSA or milk with 0.02% sodium azide and a 1:5,000 dilution of rabbit polyclonal anti-VSV antibodies (raised against VSV virions), a 1:1,000 dilution of rabbit Phospho-Stat1 (Tyr701) (Cell Signaling, Cat. No. 9167S), a 1:1,000 dilution of rabbit anti-STAT1 (Cell Signaling, Cat. No. 14994T), a 1:500 dilution of rabbit anti-METTL3 (Thermo Fisher Scientific, Cat. No. 15073-1-AP), a 1:1,000 dilution of rabbit anti-RIG-I (Cell Signaling, 4200S), a 1:1,000 dilution of rabbit anti-cGAS (Cell Signaling, 15102S), or a 1:1,000 dilution of rabbit anti-MX1 (Proteintech, Cat. No. 13750-1-AP). Starbright Blue 700 goat anti-rabbit IgG fluorescent secondary antibodies (Bio-Rad, Cat. No. 12004161) at 1:5,000 dilutions were used for fluorescent western blotting detection using the ChemiDoc MP Imaging System from Bio-Rad. Brightness and contrast were adjusted uniformly across the entire image to improve band visualization without selective enhancement, splicing, or alteration of the original data. To detect total protein, the membranes were stained with Coomassie Blue stain.

### Replication-competent viruses

The following recombinant viruses encoding GFP reporter were used: VSV (Indiana serotype) (VSV-GFP) (40), Sendai virus (SeV-GFP), and respiratory syncytial virus (RSV-GFP). SeV-GFP (SeV-GFP-Fmut) has the GFP open reading frame (ORF) at position 1 of the viral genome and a mutation in the cleavage site of the fusion (F) protein, allowing F activation and production of infectious virus particles in cells without acetylated trypsin in the medium through a ubiquitous furin-like protease (41). RSV-GFP has the GFP ORF at position 1 of the viral genome (42). VSV-GFP was grown in BHK-21 cells; SeV-GFP was grown in Vero cells; and RSV-GFP was grown in Hep-2 cells.

### Virus replication kinetics

A549 cells were plated onto 96-well plates. Transfection of A549 with 50 nM iNANPs was carried out using DOTAP as a carrier for the final volume of 100 μL. Virus was then added at MOI of 0.1 in serum-free DMEM medium and incubated for 1 h at 37°C. After 1 h of incubation, the medium was removed, and 5% FBS-containing medium was added to the cells, followed by further incubation at 37°C in 5% CO_2_ for the duration of the experiment. Virus-encoded GFP fluorescence was monitored at various time points over 114 h using a fluorescence multi-well plate reader, with GFP fluorescence readings taken at 485/530 nm on the Tecan Infinite F200.

### Cell viability assay

Following virus replication kinetics, for cell viability, WST-8 (Dojindo, CK04) was added to each well for 4 h at 37°C in 5% CO_2_, then read at 450 nm using a multi-well plate reader.

### Cytokine array

A549 cells were plated in a 24-well plate at 80,000 cells/well density in 450 μL per well. Transfection of A549 with 50 nM iNANPs was carried out using DOTAP as a carrier for the final volume of 500 μL. Virus was then added at MOI of 0.1 in a serum-free DMEM medium and incubated for 1 h at 37°C. After 1 h of incubation, the medium was removed, and 5% FBS-containing medium was added to the cells. After 20 h, cell supernatants (for cytokine array) and lysates (for western blot) were collected and stored at −80°C. Supernatants were then sent to EVE Technologies for the Human Interferon 9-Plex Discovery Assay Array (Eve Technologies, Cat. No. HDIFN9).

### Cell cytotoxicity assay

A549, SUIT-2, and AG16404 cells were plated onto 96-well plates. Transfection with 50 nM RNA cubes or 50 ng/μL poly(I:C) was carried out using DOTAP as a carrier for the final volume of 100 μL. Subsequently, 28 h post-transfection, 100 μL of each condition was collected and mixed with the cytotoxicity reagent in a flat white 96-well plate at a 1:1 ratio to measure cell cytotoxicity using the CytoTox-Glo Cytotoxicity Assay (Promega, G9290). After a 20-min incubation at room temperature, the luminescence was measured using a luminometer (ns, not significant; *P < 0.05, **P < 0.01, ***P < 0.001, and ****P < 0.0001).

## Data Availability

The authors agree that any materials and data that are reasonably requested by others will be available from a publicly accessible collection or will be made available in a timely fashion, at reasonable cost, and in limited quantities to members of the scientific community for noncommercial purposes.

## IMPORTANCE

Establishing a rapid, broad-spectrum antiviral state in human cells offers a promising strategy to combat viral infections, especially when vaccines or pathogen-specific treatments are unavailable. Here, we evaluate immunostimulatory nucleic acid nanoparticles (iNANPs) identified as potent innate immune activators for their ability to induce protective antiviral states. The highly modular design and tunable physicochemical properties of iNANPs with defined architectures and compositions can be tailored to engage specific innate immune sensors. This same modularity allows a seamless “plug-and-play” integration of diverse therapeutic nucleic acids, as well as other therapeutics and small molecules, directly into the iNANP structure. We tested iNANPs for antiviral efficacy against a replication-incompetent lentiviral vector pseudotyped with vesicular stomatitis virus (VSV) G protein, as well as replication-competent viruses, including VSV, Sendai virus (SeV), and respiratory syncytial virus (RSV). Our results demonstrate that iNANPs significantly restrict viral infection, highlighting their potential as a broad-spectrum antiviral prophylactic platform.
